# Supplementary material for: Complex‐centric proteome profiling by SEC‐SWATH‐MS
Source: Mol Syst Biol. 2019 Jan 14;15(1):e8438. doi: 10.15252/msb.20188438 (PMC6346213; doi:10.15252/msb.20188438)
Supplement: Supplementary file 7 — Dataset EV6 [file MSB-15-e8438-s007.zip › feature_plots_bioplex/Q9UI38.pdf]

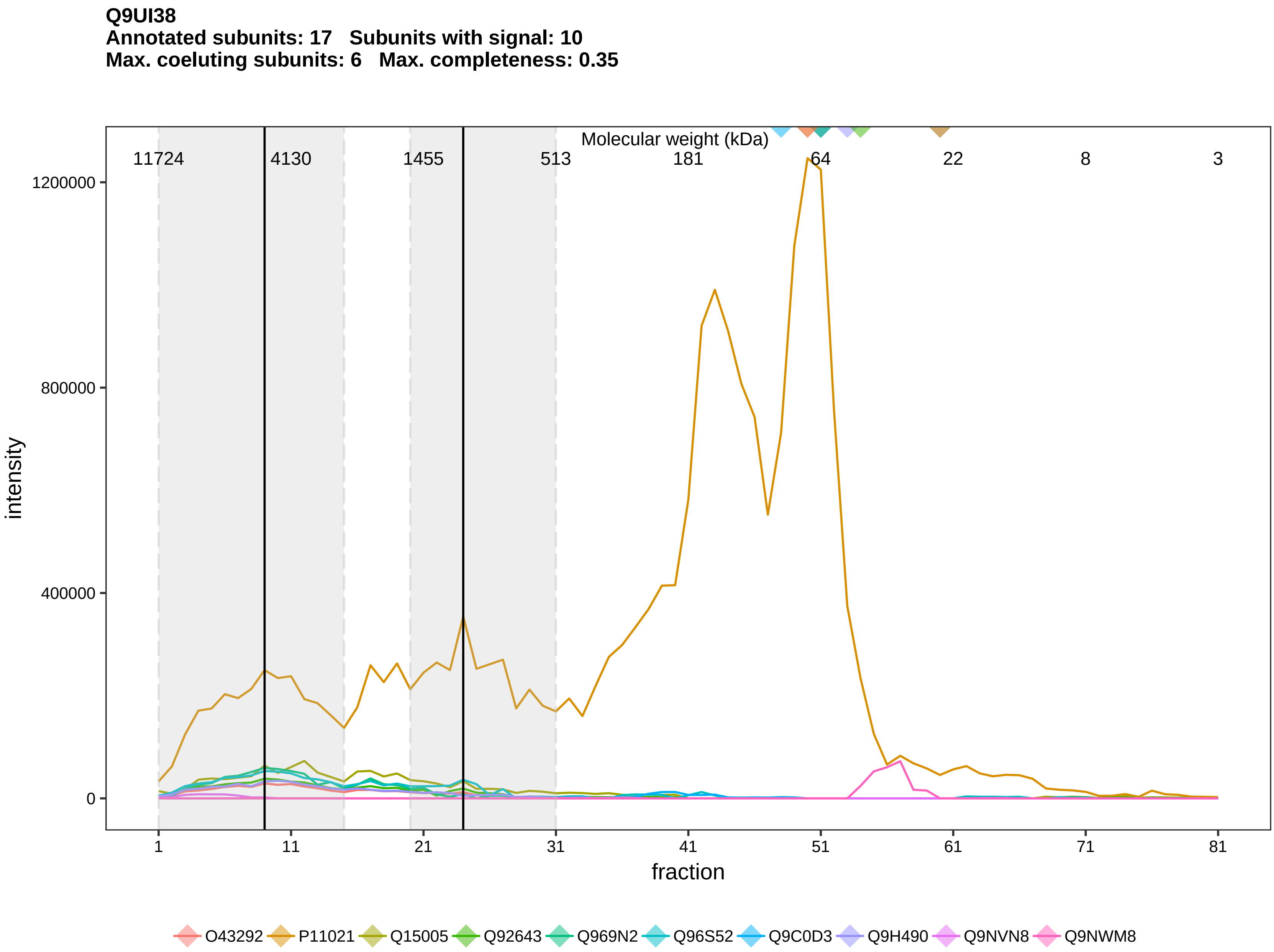

Q9UI38
Annotated subunits: 17 Subunits with signal: 10
Max. coeluting subunits: 6 Max. completeness: 0.35
Molecular weight (kDa)
11724
4130
1455
513
181
64
22
8
3
1200000
800000
400000
0
intensity
1
11
21
31
41
51
61
71
81
fraction
O43292
P11021
Q15005
Q92643
Q969N2
Q96S52
Q9C0D3
Q9H490
Q9NVN8
Q9NWM8
